# Vorinostat positively regulates synaptic plasticity genes expression and spine density in HIV infected neurons: role of nicotine in progression of HIV-associated neurocognitive disorder

**DOI:** 10.1186/1756-6606-7-37

**Published:** 2014-05-15

**Authors:** Venkata Subba Rao Atluri, Sudheesh Pilakka-Kanthikeel, Thangavel Samikkannu, Vidya Sagar, Kesava Rao Venkata Kurapati, Shailendra K Saxena, Adriana Yndart, Andrea Raymond, Hong Ding, Oscar Hernandez, Madhavan PN Nair

**Affiliations:** 1 Department of Immunology, Institute of NeuroImmune Pharmacology, Herbert Wertheim College of Medicine, Florida International University, 11200 SW 8th Street, Miami, FL -33199, USA; 2 CSIR- Centre for Cellular and Molecular Biology, Uppal Road, Hyderabad, India

**Keywords:** Human synaptic plasticity, PCR array, Spine density, HIV, Nicotine, HDAC2, eIF2α, Vorinostat, SK-N-MC neuronal cells, Neurocognitive disorder

## Abstract

**Background:**

HIV-associated neurocognitive disorder (HAND) is characterized by development of cognitive, behavioral and motor abnormalities, and occurs in approximately 50% of HIV infected individuals. In the United States, the prevalence of cigarette smoking ranges from 35-70% in HIV-infected individuals compared to 20% in general population. Cognitive impairment in heavy cigarette smokers has been well reported. However, the synergistic effects of nicotine and HIV infection and the underlying mechanisms in the development of HAND are unknown.

**Results:**

In this study, we explored the role of nicotine in the progression of HAND using SK-N-MC, a neuronal cell line. SK-N-MC cells were infected with HIV-1 in the presence or absence of nicotine for 7 days. We observed significant increase in HIV infectivity in SK-N-MC treated with nicotine compared to untreated HIV-infected neuronal cells. HIV and nicotine synergize to significantly dysregulate the expression of synaptic plasticity genes and spine density; with a concomitant increase of HDAC2 levels in SK-N-MC cells. In addition, inhibition of HDAC2 up-regulation with the use of vorinostat resulted in HIV latency breakdown and recovery of synaptic plasticity genes expression and spine density in nicotine/HIV alone and in co-treated SK-N-MC cells. Furthermore, increased eIF2 alpha phosphorylation, which negatively regulates eukaryotic translational process, was observed in HIV alone and in co-treatment with nicotine compared to untreated control and nicotine alone treated SK-N-MC cells.

**Conclusions:**

These results suggest that nicotine and HIV synergize to negatively regulate the synaptic plasticity gene expression and spine density and this may contribute to the increased risk of HAND in HIV infected smokers. Apart from disrupting latency, vorinostat may be a useful therapeutic to inhibit the negative regulatory effects on synaptic plasticity in HIV infected nicotine abusers.

## Background

HIV-1 infection, cigarette smoking and drug abuse are global public health concerns. Several studies report that nicotine, the psychoactive drug in tobacco products that induces dependence among smokers, is one of the major immunomodulatory agents [1-5]. Nicotine dependence is the most common form of chemical dependence in the United States. Research suggests that nicotine may be as addictive as heroin, cocaine and alcohol [3,5]. In the United States, the prevalence of cigarette smoking ranges from 35-70% in HIV-infected individuals compared to approximately 20% in general population [6-9].

Infection with HIV is often associated with a wide range of neurological and neuropathological abnormalities, which are collectively known as HIV- associated neurocognitive disorder (HAND). HAND is characterized by development of cognitive, behavioral and motor abnormalities, and occurs in approximately 50% of HIV infected individuals [10,11]. Previous studies indicate that HIV-1 clade B (predominant subtype found in the United States and in the western world) being more neuropathogenic than clade C (predominant in sub-Saharan Africa and Asia) [12-14]. Experimental evidence and clinical observations suggest a correlation between smoking and increased risk for neurological disorders [15]. A great deal of research has focused on the pathophysiological and clinical effects of nicotine. In human studies, nicotine and other agonists of the nicotinic acetylcholine receptor (nAChRs) have been reported to improve cognitive performance in healthy adults and in different psychiatric populations [16,17]. In studies with rodents, the results have been mixed with observations of improvement, impairment, or no effect on various measures of learning and memory following acute or chronic systemic treatment [16]. However, the effect of nicotine on HIV-1 pathogenesis has received limited research attention. Few studies have reported that nicotine exacerbates HIV-1 replication in in vitro-infected alveolar macrophages and primary human microglia [18,19]. Further, limited examination of the neuropathogenic effects of nicotine on HIV-1 infection of CNS cells has shown that galantamine and nicotine exert synergistic effect on the inhibition of microglial cell activation induced by HIV-1 gp120 [20]. A recent study reported that exposure to tobacco smoke extract increases the blood–brain barrier permeability and plays a critical role in pathophysiology of ischemia [21].

Although few studies have reported deficits in learning, memory, and global cognitive performance among cigarette smokers living with HIV [22,23], the underlying mechanisms associated with the use of nicotine and the development of HAND in these patients are unknown. New memory formation involves gene expression-dependent changes in synaptic structure and plasticity in the hippocampus [24]. Recently, we have reported dysregulated synaptic plasticity genes expression and decreased spine density in HIV infected CNS cells [13]. Although epigenetic modifications are known to regulate gene expression during HIV infection [25], molecular and biochemical evidence on epigenetic mechanisms in CNS cells in response to HIV and nicotine are lacking. Acetylation and deacetylation of histone proteins associated with chromatin plays a pivotal role in the epigenetic regulation of transcription and other functions in cells, including neurons.

**Figure 1:**
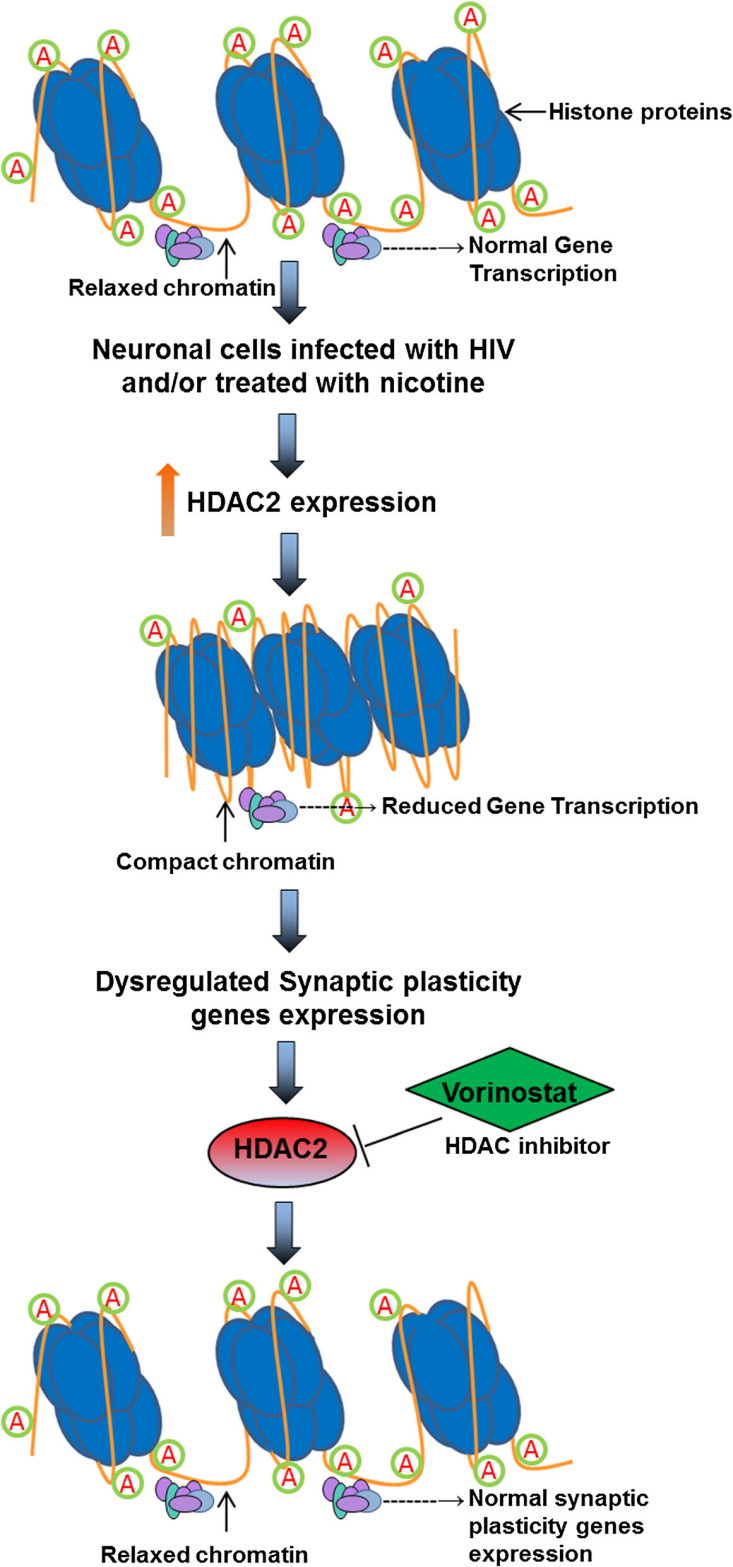
**A hypothetical model for the role of HDAC2 in transcriptional repression of synaptic plasticity genes in neuronal cells infected with HIV and/or in the presence of nicotine.** During the gene transcription, the DNA to be transcribed is associated with histone proteins (blue) that are modified by the addition of acetyl groups (green). This modification results in a relaxed chromatin configuration that allows the transcriptional machinery access to the DNA. Up-regulation of HDAC2 during HIV infection and/or nicotine treatment leads to deletion of acetyl groups from histone proteins, resulting in a condensed chromatin that limits the binding of the transcriptional machinery, thereby decreasing gene transcription. Thus, inhibition of HDAC2 by using vorinostat may block these enzymatic processes and return the chromatin to a relaxed state, allowing gene transcription.

**Figure 2:**
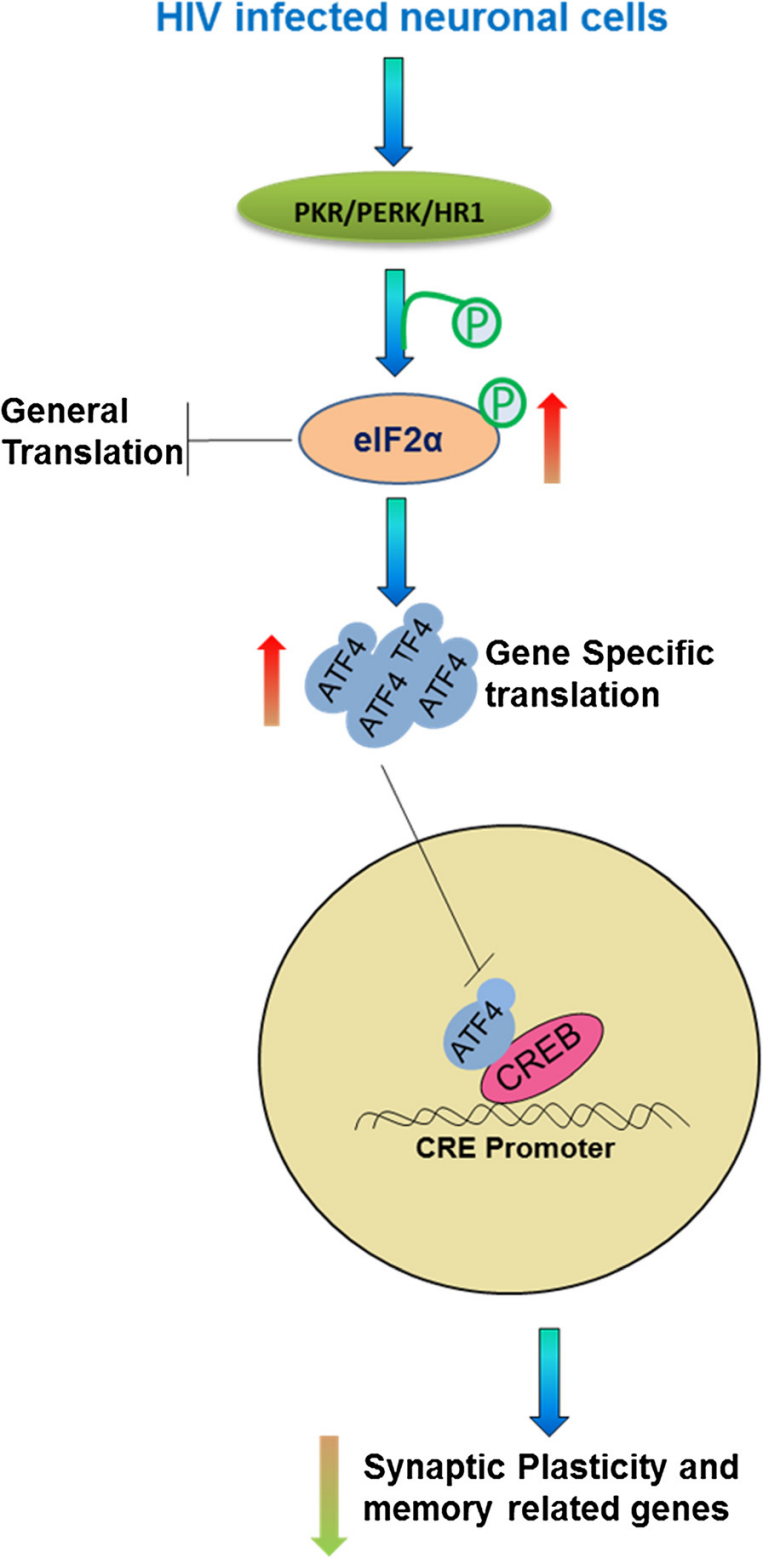
**A hypothetical model for the role of eIF2α phosphorylation in translational repression of synaptic plasticity genes in HIV infected neuronal cells.** In HIV infection, due to partial phosphorylation of eIF2α, general translation is reduced and ATF4 mRNA translation is augmented. As a consequence, the expression of synaptic plasticity and memory-related genes is blocked.

HDACs often function as a component of the transcriptional repressor complex to silence gene expression and induce chromatin compaction through histone protein deacetylation. HDAC2 functions in modulating synaptic plasticity and long-lasting changes of neural circuits, which in turn negatively regulates learning and memory [26]. HDAC inhibitors are emerging as an exciting new class of potential therapeutic agents for the treatment of solid and hematological malignancies. Vorinostat also known as suberanilohydroxamic acid (SAHA) was the first histone deacetylase inhibitor approved by the U.S. Food and Drug Administration for the treatment of Cutaneous T-Cell Lymphoma. Vorinostat binds to the catalytic domain of the histone deacetylases (HDACs). This allows the hydroxamic moiety to chelate zinc ion located in the catalytic pockets of HDAC, thereby inhibiting deacetylation and leading to an accumulation of both hyperacetylated histones and transcription factors. Vorinostat was also shown to cross the blood–brain barrier in a mouse model of Huntington's disease [27]. Therefore, use of vorinostat as a therapeutic agent in the CNS related diseases may be useful. In the down-stream, translation of eukaryotic mRNAs is regulated primarily at the level of initiation. Two main mechanisms by which translation is controlled are the formation of the ternary complex via eIF2 and the recruitment of the ribosome to the mRNA via 4E-BPs. Phosphorylation of an α subunit of eIF2 (at Ser51) converts the protein from a substrate to a competitive inhibitor of the GDP/GTP-exchange reaction by decreasing the rate of dissociation of eIF2 from eIF2B [28,29]. This causes a decrease in general translational initiation [29,30]. We hypothesize that by inducing the transcriptional repression, HDAC2 plays a major role in the neuropathogenesis caused by HIV and nicotine (Figure 1). In the down-stream, we hypothesize that translational process in HIV infected cells will be affected by eIF2α phosphorylation (Figure 2).

In the current study, we have analyzed the effect of nicotine on the differential expression of synaptic plasticity genes, spine density in an in vitro HIV infection model using neuronal cells. Along with the dysregulated synaptic plasticity genes and altered spine density, we have found increased levels of HDAC2 expression indicating the transcriptional repression of synaptic plasticity genes in these cells. We also found increased eIF2α subunit phosphorylation (at Ser51) which may associate with the translational repression of synaptic plasticity genes in HIV alone infected SK-N-MC cells and in the presence of nicotine. These negative regulatory effects of HIV infection and nicotine on the expression of synaptic plasticity genes and spine density were reversed by the HDAC inhibitor vorinostat. This study suggests that nicotine synergistically acts with HIV to down-regulate synaptic plasticity genes and alter spine density, potentially via upregulation of HDAC2. The use of vorinostat may help to reverse these negative regulatory effects (induced by HDAC2) on synaptic plasticity genes expression and spine density.

## Results

### Increased HIV-infectivity rate of neuronal cells in the presence of nicotine

**Figure 3:**
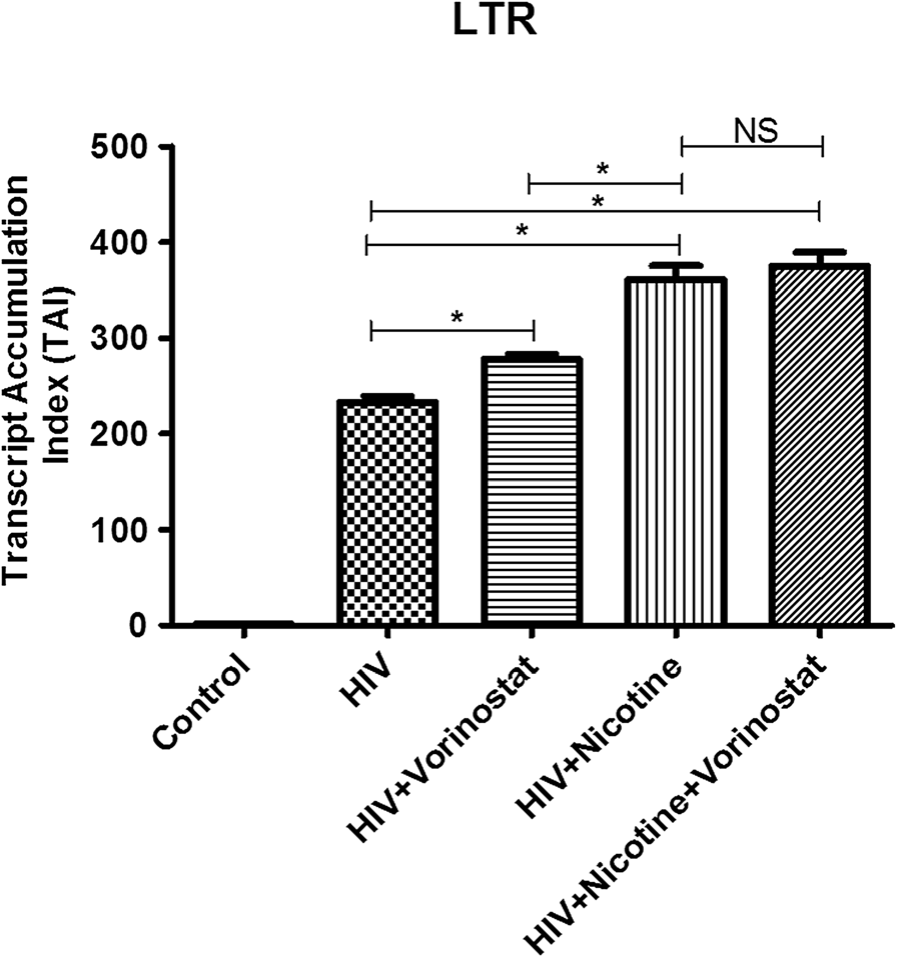
**Increased HIV-infectivity rate of neuronal cells in the presence of nicotine.** SK-N-MC cells were infected with HIV for 7 days with/without nicotine. Cells were harvested; RNA was isolated from the infected cells. LTR real-time PCR analysis indicated that HIV infectivity rate was significantly higher in the presence of nicotine than HIV alone infected cells. Significantly increased HIV infectivity (latency disruption) in vorinostat treated neuronal cells was also observed (not significant in combination with nicotine). (*, p ≤ 0.05; NS-Not Significant).

We observed significant increase in HIV-infection in nicotine-treated HIV-infected SK-N-MC compared to the HIV- infected alone cells by using the LTR real-time PCR. We also observed significantly increased HIV infection (latency disruption) in HIV infected neuronal cells in the presence of vorinostat by both LTR real-time PCR (Figure 3) and p24 ELISA (data not shown). We did not see significant levels of vorinostat induced latency disruption in HIV infected neuronal cells in the presence of nicotine and vorinostat.

### Differential expression of human synaptic plasticity genes in SK-N-MC cells infected with HIV and/or in the presence of nicotine

**Figure 4:**
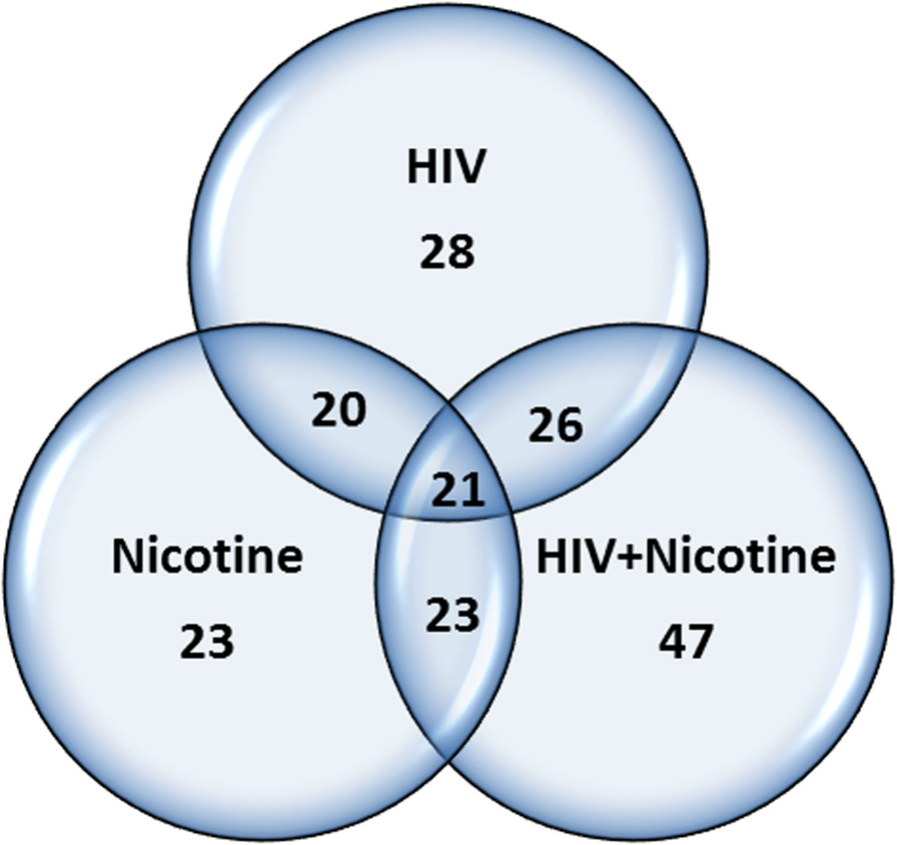
Venn diagram comparing the number of down-regulated synaptic plasticity genes in SK-N-MC neuronal cells infected with HIV and/or in the presence of nicotine.

Out of 84 human synaptic plasticity genes expression analyzed by human synaptic plasticity PCR array, 23 synaptic plasticity genes were significantly down-regulated whereas 16 synaptic plasticity genes were significantly up-regulated in nicotine treated SK-N-MC cells. In the HIV infected SK-N-MC cells, while 28 synaptic plasticity genes were down-regulated, 8 synaptic plasticity genes were significantly up-regulated. However, in the presence of nicotine, in HIV infected SK-N-MC cells, a total of 47 genes were significantly down-regulated (3–22 fold) and 4 genes were significantly up-regulated (Table 1). Out of the total 84 genes, 21 genes were commonly down regulated in all the three groups (nicotine treated, HIV infected and HIV along with nicotine). Figure 4 (Venn diagram) shows the number of down-regulated synaptic plasticity genes in SK-N-MC neuronal cells infected with HIV and/or nicotine combination. These results indicate that in the presence of nicotine, dysregulation of synaptic plasticity genes will be further increased in HIV infected neuronal cells.

**Table 1:** Human synaptic plasticity genes expression in HIV infected neuronal cells and in combination with nicotine and/or vorinostat (fold change)

***Genes***	**Vorinostat**	**HIV**	**HIV + Vorinostat**	**Nicotine**	**Nicotine + Vorinostat**	**HIV + Nocotine**	**HIV + Nicotine + Vorinostat**
*ADAM10*^*5,6,9*^	1.47	1.89	1.24	**4.12**	1.45	-1.03	1.11
*ADCY1*^*3*^	1.12	**-3.39**	1.12	**-3.15**	2.26	**-3.99**	1.17
*ADCY8*^*3*^	1.12	**-3.39**	1.12	**-3.15**	2.26	**-3.99**	-1.96
*AKT1*^*7*^	**3.10**	2.96	**3.84**	**3.42**	**4.59**	-1.32	**4.23**
*ARC*^*1,9*^	-1.09	1.30	1.54	1.07	2.79	**-4.81**	1.55
*BDNF*^*1,3*^	1.12	**-3.39**	1.12	**-3.15**	2.26	**-3.99**	-1.96
*CAMK2A*^*3*^	1.12	**-3.39**	1.12	**-3.15**	2.26	**-3.99**	-1.96
*CAMK2G*^*3,7*^	1.48	1.11	1.46	1.16	1.62	**-3.38**	1.33
*CDH2*^*3,5*^	1.12	**-3.06**	1.12	**-3.15**	2.26	**-3.99**	-1.37
*CEBPB*^*1*^	1.37	2.50	1.64	1.98	2.07	**-3.07**	2.04
*CEBPD*^*1*^	1.39	1.89	1.96	1.35	**3.03**	**-3.07**	1.96
*CNR1*^*3*^	1.12	**-3.39**	1.12	**-3.15**	2.26	**-3.99**	-1.96
*CREM*^*1*^	**-35.90**	**-9.78**	**-15.78**	**-8.26**	**-16.02**	**-25.41**	**-16.77**
*EGR1*^*1*^	2.37	1.13	1.34	**-5.68**	1.15	**-9.24**	1.20
*EGR2*^*1*^	**6.89**	1.94	**4.29**	**9.22**	**3.34**	1.43	2.43
*EGR3*^*1*^	1.86	1.01	**3.89**	-1.76	2.94	**-3.99**	2.43
*EGR4*^*1*^	-1.95	**-6.69**	-1.96	**-3.20**	1.03	**-8.74**	**-4.28**
*EPHB2*^*8*^	1.71	-2.97	1.74	**-3.15**	2.36	**-3.99**	1.43
*FOS*^*1*^	1.15	-1.24	1.07	1.07	1.43	**-3.83**	-1.12
*GABRA5*^*3,8*^	1.91	**-3.39**	1.12	-1.62	2.26	**-3.99**	-1.96
*GRIA1*^*3,4,8,9*^	1.12	**-3.39**	1.12	**-3.15**	2.26	**-3.99**	-1.96
*GRIA2*^*3,4,8*^	**4.04**	2.38	2.55	2.09	**4.40**	1.10	**3.49**
*GRIA4*^*4,8,9*^	**12.85**	**9.24**	**4.35**	2.39	**5.73**	-1.50	**3.12**
*GRIN1*^*3,7,8,9*^	**31.65**	**5.89**	**28.25**	**4.70**	**39.89**	-1.02	**35.80**
*GRIN2A*^*3,5,7,8,9*^	2.26	**-3.06**	1.12	-2.14	2.26	**-3.99**	-1.07
*GRIN2B*^*3,5,7,8,9*^	2.26	**-3.39**	1.12	**-3.15**	2.26	**-3.99**	-1.07
*GRIN2C*^*3,7,8,9*^	2.26	-1.73	1.80	**-3.15**	2.26	**-3.99**	-1.07
*GRIN2D*^*3,7,8*^	**4.25**	1.42	2.17	**4.97**	**4.25**	-1.34	2.80
*GRIP1*^*4*^	**3.07**	-1.09	2.36	2.72	**3.07**	-1.90	1.96
*GRM1*^*4,8,9*^	1.12	**-3.39**	1.12	**-3.15**	2.26	**-3.99**	-1.96
*GRM2*^*4,8*^	1.85	1.93	1.12	**3.79**	2.26	-1.47	1.01
*GRM3*^*8,9*^	1.12	**-3.39**	1.12	-2.11	2.26	**-3.99**	-1.96
*GRM4*^*8*^	2.26	**-3.06**	1.12	1.34	2.26	**-3.99**	-1.21
*GRM5*^*8*^	1.12	**3.38**	1.12	-1.15	2.26	-2.75	-1.96
*GRM7*^*8*^	1.12	**-3.06**	1.12	2.45	2.26	-2.33	-1.10
*HOMER1*^*1,9*^	-1.64	-1.13	-2.95	1.30	-2.68	-1.93	**-3.13**
*IGF1*^*4*^	1.96	**9.13**	1.32	**11.75**	1.19	**3.37**	1.17
*INHBA*^*2*^	**4.15**	-2.11	2.03	-1.43	2.22	**-5.89**	2.25
*JUN*^*1*^	**8.84**	-1.39	**6.73**	-1.65	**8.04**	**-11.61**	**6.33**
*JUNB*^*1*^	**3.93**	2.73	**4.06**	1.42	**10.84**	**-3.97**	**3.79**
*KIF17*^*10*^	2.30	-2.99	**3.20**	-1.89	**4.19**	**-9.30**	**3.34**
*MMP9*^*1,3,6*^	**3.06**	-2.68	2.00	-2.94	2.99	**-10.32**	1.62
*NCAM1*^*5*^	1.17	**-4.59**	1.41	**-6.80**	1.40	**-39.88**	1.25
*NFKB1*^*1*^	**-20.48**	**-6.41**	**-35.26**	**-5.45**	**-26.76**	**-25.59**	**-48.77**
*NFKBIB*^*1*^	**9.61**	**12.91**	**13.09**	**14.56**	**14.20**	**13.54**	**13.66**
*NGF*^*1,4*^	**5.79**	1.45	**4.92**	1.30	**4.72**	**-3.01**	**6.55**
*NGFR*^*4*^	1.30	2.50	1.13	2.65	1.37	-2.18	1.04
*NOS1*^*4*^	1.12	**-3.39**	1.12	1.22	2.26	**-3.99**	-1.96
*NPTX2*^*1*^	1.12	2.22	1.12	1.28	2.26	**-3.29**	**3.19**
*NR4A1*^*1*^	**5.11**	1.47	**6.73**	2.54	**4.82**	-1.79	**4.67**
*NTF3*^*1*^	1.12	**-3.06**	1.12	1.92	2.26	-1.53	-1.96
*NTF4*^*3*^	1.98	1.14	**4.82**	-1.30	**5.85**	**-6.14**	**4.63**
*NTRK2*^*3*^	1.12	**-3.40**	1.12	**-3.15**	2.26	**-3.99**	-1.96
*PCDH8*^*1,5*^	2.37	2.53	1.29	**3.37**	2.04	-1.68	1.35
*PICK1*^*4,9*^	1.58	1.54	2.64	1.18	2.65	**-4.21**	2.30
*PIM1*^*1*^	**11.19**	**4.86**	**25.46**	**10.59**	**33.08**	2.55	**32.95**
*PLAT*^*1,4,6*^	-1.03	-1.82	-1.03	-2.34	1.97	**-4.59**	-2.25
*PLCG1*^*3*^	2.19	1.23	2.35	1.39	**3.78**	**-3.62**	2.91
*PPP1CA*^*3,4,7*^	-2.04	1.54	1.37	-1.69	1.16	**-13.25**	1.48
*PPP1R14A*^*4*^	1.12	**-3.06**	1.12	**-3.15**	2.26	**-3.99**	-1.96
*PPP2CA*^*4*^	**6.52**	**10.06**	**6.87**	**13.31**	**7.20**	**3.28**	**6.64**
*PRKCA*^*3,4*^	**5.40**	2.80	**3.29**	**7.23**	**4.43**	2.24	2.93
*PRKCG*^*3*^	2.30	**-5.07**	2.16	**-4.84**	**3.22**	**-6.62**	2.12
*PRKG1*^*4*^	1.12	**-3.06**	1.12	**-3.15**	2.26	**-3.99**	-1.47
*RAB3A*^*3*^	**86.46**	**56.49**	**106.15**	**75.27**	**91.65**	**15.81**	**103.39**
*RELA*^*1*^	1.30	2.00	1.34	**3.26**	1.45	-1.12	1.25
*RELN*^*5,6*^	1.12	**-3.06**	1.12	**-3.15**	2.26	**-3.99**	-1.96
*RGS2*^*1*^	**5.29**	-1.40	2.62	-1.12	**4.46**	-2.90	2.38
*RHEB*^*1*^	**-49.73**	**-32.90**	**-57.68**	**-35.16**	**-46.92**	**-84.30**	**-69.94**
*SIRT1*^*10*^	2.29	2.58	1.91	**4.70**	1.89	1.52	1.28
*SRF*^*1*^	2.70	2.83	1.82	**3.19**	2.33	-1.44	1.60
*TIMP1*^*6*^	**-18.20**	**-38.00**	**-20.11**	**-24.02**	**-15.69**	**-79.20**	**-17.73**
*TNF*^*1,5*^	-2.65	**-5.47**	-2.28	-2.63	-1.42	**-5.97**	**-9.30**

Out of 84 genes analyzed, only genes significantly (± ≥ 3 fold) dysregulated were shown in this table.

^1^Immediate Early Response Genes; ^2^Late Response Genes; ^3^Long Term Potentiation; ^4^Long Term Depression; ^5^Cell Adhesion; ^6^Extracellular Matrix & Proteolytic Processing; ^7^CREB Cofactors; ^8^Neuronal Receptors; ^9^Postsynaptic Density; ^10^Others.

If the synaptic plasticity genes expression change is ± ≥3 fold, the fold change was represented in bold letters.

### Vorinostat regulates HDAC2 expression in neuronal cells infected with HIV and/or treated with nicotine

**Figure 5:**
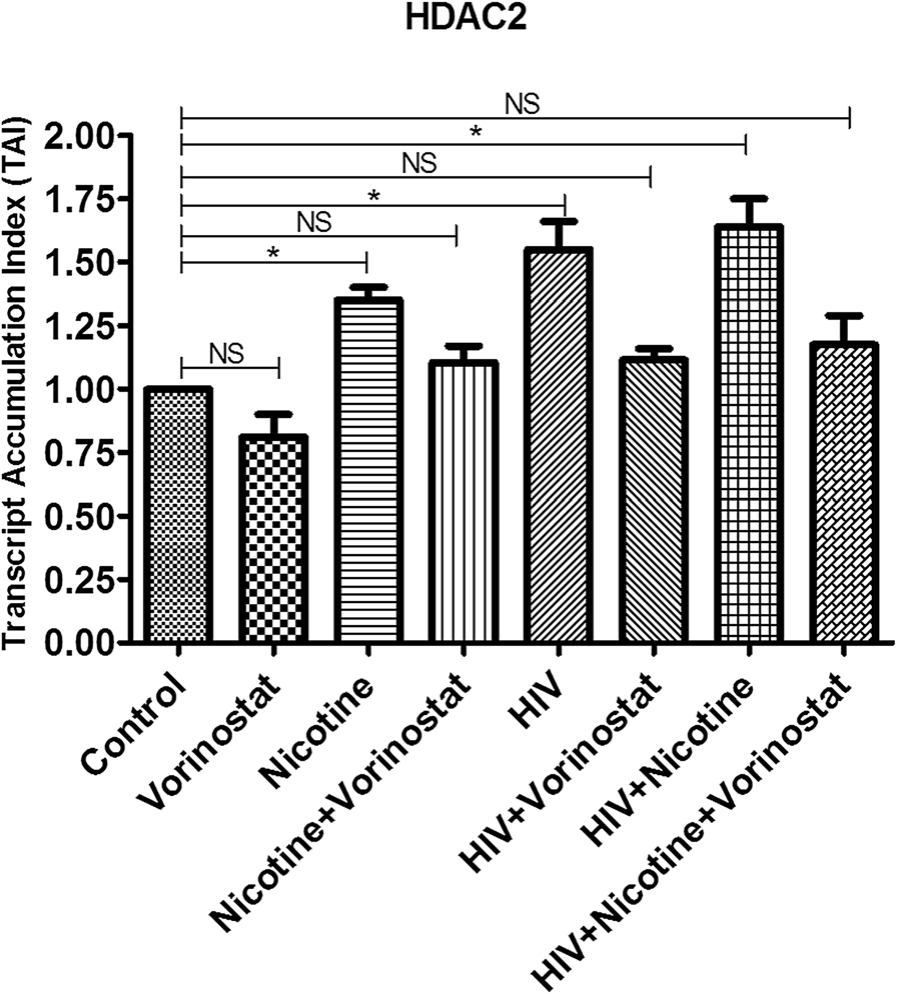
**qPCR analysis for HDAC2 expression in SK-N-MC cells infected with HIV and/or treated with nicotine/vorinostat combination.** SK-N-MC cells were infected with HIV and/or treated with nicotine. On the 5th day, 1 μg of vorinostat was added to the nicotine treated, HIV infected, HIV + nicotine treated cells. After total 7 days of HIV infection and/or treatment with nicotine, cells were harvested; RNA was isolated from the cells using illustra triplePrep Kit. The qPCR analysis indicated significantly increased HDAC2 expression in nicotine treated; HIV infected and in combined treatment. Vorinostat could able to inhibit the HDAC2 up-regulation in all the vorinostat treated test groups. (*, p ≤ 0.05; NS-Not Significant).

**Figure 6:**
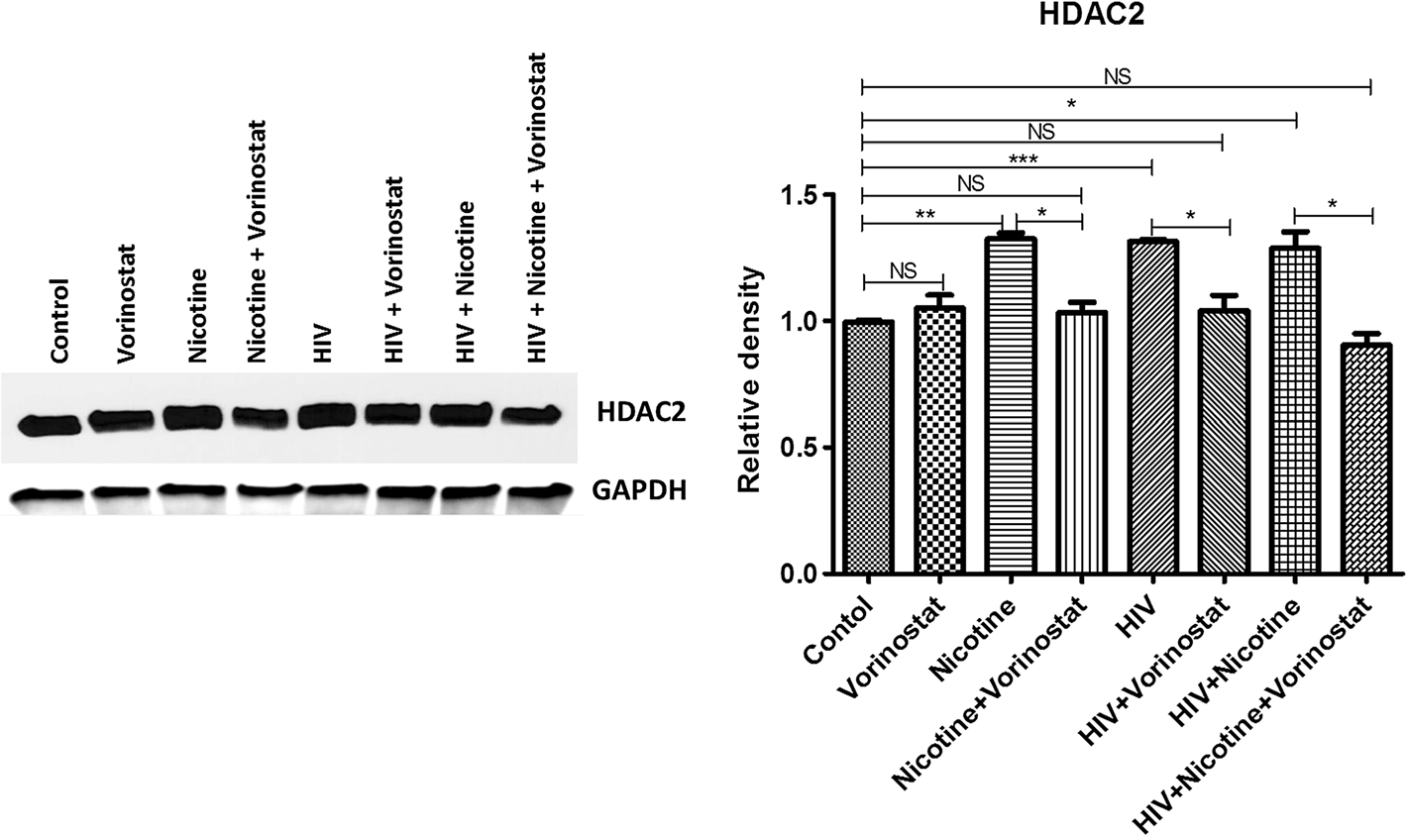
**Western blot analysis for HDAC2 expression in SK-N-MC cells infected with HIV and/or treated with nicotine/vorinostat combination.** SK-N-MC cells were infected with HIV and/or treated with nicotine. On the 5th day, 1 μg of vorinostat was added to the nicotine treated, HIV infected, HIV + nicotine treated cells. After total 7 days of HIV infection and/or treatment with nicotine, cells were harvested; protein was isolated from the cells using illustra triplePrep Kit. Transfer blots were probed with anti-HDAC2 antibody followed by secondary HRP conjugated antibody. Western blot results support the qPCR results indicating that HDAC2 up-regulation can be inhibited by vorinostat (at 1 μg conc.) in HIV and/or nicotine treated neuronal cells. (*, p ≤ 0.05; **, p ≤ 0.01; ***, p ≤ 0.001; NS-Not Significant).

Using qPCR (Figure 5) and western blot analysis (Figure 6), we observed significantly higher HDAC2 levels in nicotine treated cells, HIV infected cells and HIV infected cells treated with nicotine compared to control cells, indicating possible epigenetic changes which are responsible for the transcriptional repression of synaptic plasticity genes. But we did not see additive/synergistic effect on HDAC2 expression in HIV infected cells in the presence of nicotine. Vorinostat is a potent inhibitor of Classes I and II histone deacetylases. To inhibit the up-regulation of HDAC2 expression in SK-N-MC cells infected with HIV and/or treated with nicotine, these test groups were treated with vorinostat on the 5th day of infection and/or nicotine treatment. Using vorinostat (at 1 μM conc.), we were able to inhibit the HDAC2 expression levels in the nicotine treated cells and HIV infected neuronal cells with/without nicotine to the expression levels approximately equal to the control cells (Figures 5 and 6).

### Vorinostat positively regulates synaptic plasticity genes expression in HIV infected and/or nicotine treated neuronal cells

**Figure 7:**
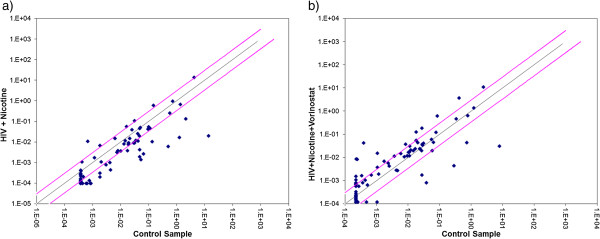
**Representative figures for scatter plot analysis of the changes in synaptic plasticity gene expression in HIV infected SK-N-MC neuronal cells with nicotine and in combination with vorinostat.** Pair wise comparison of control SK-N-MC cells (No HIV infection) and **a)** HIV + Nicotine **b)** HIV + Nicotine + Vorinostat treated SK-N-MC cells by scatter plot analysis. Spots associated with individual human synaptic plasticity gene were collected and converted into log10 scale. The central line indicates unchanged gene expression. The synaptic plasticity genes with expression levels higher or lower in treated neuronal cells than control cells are expected to produce dots that deviate from the centerline. The dots are allocated to positions that are above or below than the +3 fold or -3 fold line when the differences are greater than three folds.

In vorinostat alone treated SK-N-MC cells, total 19 synaptic plasticity genes were significantly up-regulated while only 4 genes were significantly down-regulated. 24 synaptic plasticity genes out of 28 genes, which were down-regulated with HIV infection, were recovered and 16 more genes were significantly up-regulated in the presence of vorinostat. Further, in the case of HIV infected cells treated with nicotine, out of 47 down-regulated genes, expression of 40 genes were recovered upon vorinostat treatment and 15 more genes were up-regulated. Surprisingly, four genes down-regulated (CREM, NFKB1, RHEB, TIMP1) in the presence of vorinostat alone were consistently down-regulated in all the groups containing vorinostat. Excluding these four genes, vorinostat failed to recover the expression of EGR4, HOMER1 and TNF in HIV infected cells in the presence of nicotine (Table 1 and Figure 7). These results indicate that vorinostat can inhibit the negative regulatory effects on synaptic plasticity genes expression (mediated by increased HDAC2 expression) in HIV infected cells in the presence of nicotine.

### Synergistic decrease of spine density in HIV infected neuronal cells in the presence of Nicotine

**Figure 8:**
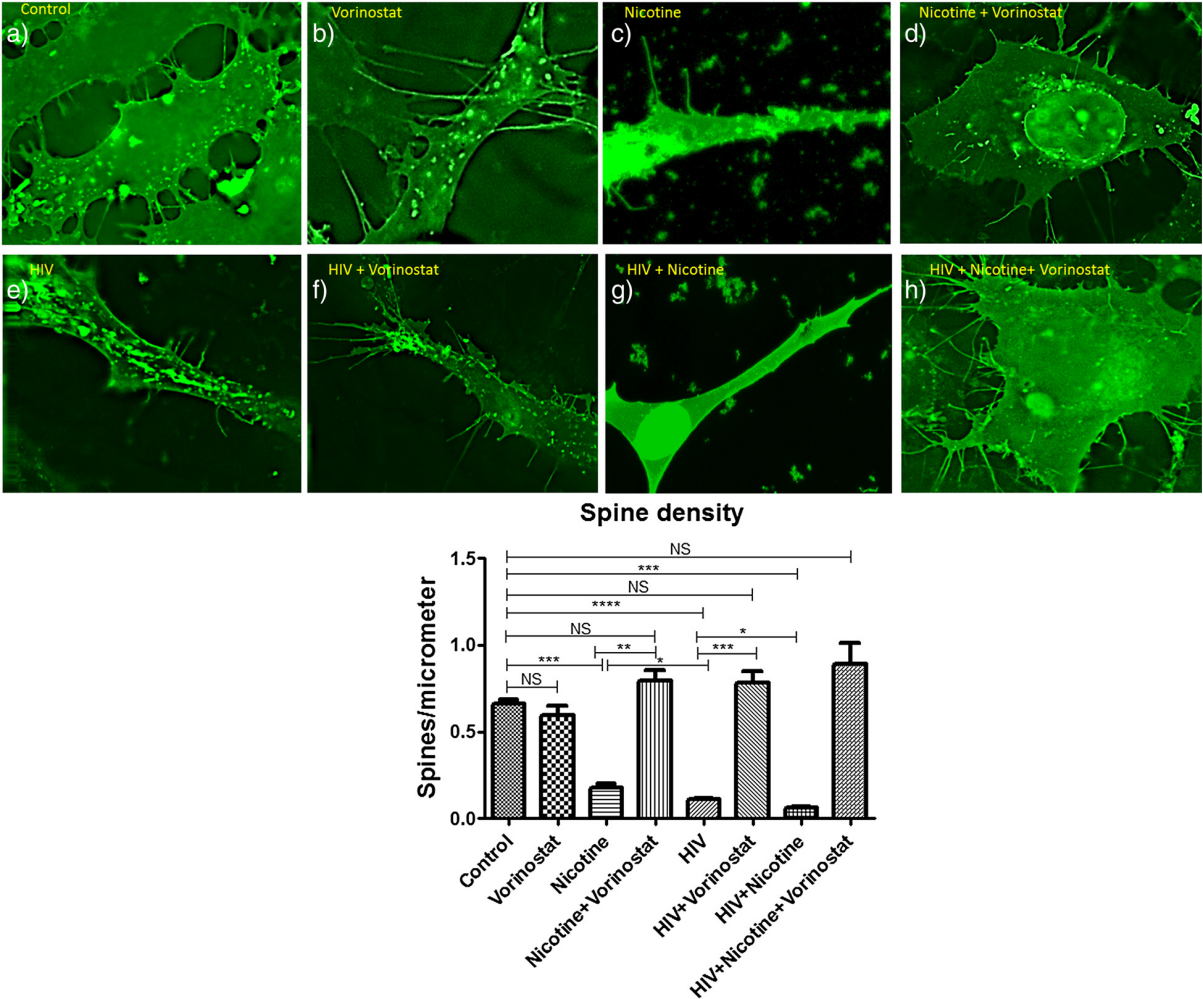
**Synergistic decrease of spine density in HIV infected neuronal cells in the presence of nicotine.** SK-N-MC cells were infected with HIV and/or treated with nicotine for 7 days. On the 5th day of HIV infection and/or nicotine treatment, vorinostat was added to the culture. **a)** Control SK-N-MC cells: High number of long spines on the dendritic length, high dendrite diameter, total dendrite area and spine area. **b)** Vorinostat treated Cells: Cells with the higher number of spines on a dendrite length similar to the control cells. **c)** Nicotine treated cells: Loss of spine on a dendrite length when compared to the control cells. **d)** Nicotine along with vorinostat: Restoration of spines on the dendrite length **e)** HIV infected cells: Loss of spines on the dendrite length, decreased dendrite diameter, dendrite and spine area was observed than the control neuronal cells. **f)** HIV infected cells in the presence of vorinostat: Recovery of spines on the dendrite length was observed. **g)** HIV infected cells in the presence of nicotine: Synergistic loss of number of spine on a dendrite length was observed when compare to the control or nicotine treated or HIV infected cells. **h)** HIV infected cells co-treated with nicotine in the presence of vorinostat: In the presence of vorinostat, spine density was restored when compare to the HIV + nicotine treated cells. (*, p ≤ 0.05; **, p ≤ 0.01; ***, p ≤ 0.001; ****, p ≤ 0.0001; NS-Not Significant).

Using the established protocol, we have observed for change in spine density in HIV infected SK-N-MC cells and/or nicotine/vorinostat combination in comparison to the control cells [13]. In nicotine alone treated cells, spine density was significantly less than the control cells. In HIV only infected cells, spine density was significantly less than the nicotine treated cells (p < 0.02). Interestingly, HIV infected cells in the presence of nicotine exhibit decreased spine density compared to nicotine/HIV alone infected cells. Spine density was recovered when these nicotine/HIV/HIV + nicotine infected cells were treated with the vorinostat (Figure 8).

### eIF2α phosphorylation in SK-N-MC cells infected with HIV and/or treated with nicotine

**Figure 9:**
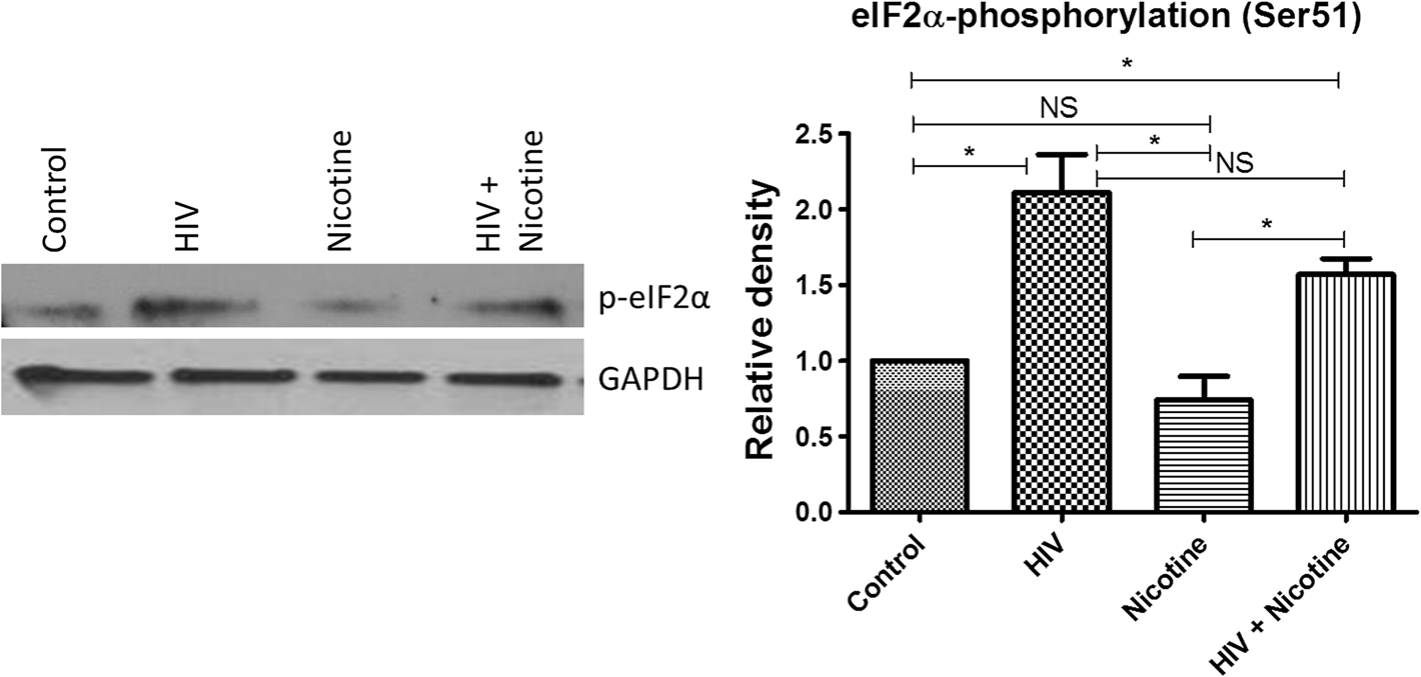
**Pattern of eIF2α phosphorylation (Ser51) in SK-N-MC neuronal cells infected with HIV alone and together with nicotine.** SK-N-MC cells were treated with HIV/nicotine alone and in combination. After 7 days of infection and/or nicotine treatment, eIF2α phosphorylation (at Ser51) was analyzed by western blot assay. We have observed significant eIF2α phosphorylation in HIV and HIV + nicotine treated groups when compared with the control cells. (*, p ≤ 0.05; NS-Not Significant).

Phosphorylation of the α-subunit of eukaryotic initiation factor 2 (eIF2α) inhibits the initiation of translational process. To investigate whether the translational process is affected or not in the HIV infected and/or nicotine treated neuronal cells, eIF2α phosphorylation (at Ser51) was analyzed. In the SK-N-MC cells, significant eIF2α phosphorylation (at Ser51) was observed in HIV infected cells alone and in combined treatment with nicotine. We did not observe significant eIF2α phosphorylation in nicotine alone treated SK-N-MC cells compared to the control cells (Figure 9).

## Discussion

Nicotine is the single tobacco constituent demonstrated to have the potential to stimulate the production of HIV-1, as has been shown using in vitro-infected alveolar macrophages [13]. A growing body of evidence suggests chronic cigarette smoking alone is associated with abnormalities in brain morphology and neurophysiology [31,32]. Reports on effect of nicotine on HIV associated neurocognitive disorders are scanty. We have used SK-N-MC cells to study the HIV infectivity and for the analysis of synaptic plasticity genes expression and spine density changes as these cells have been reported to be susceptible for the productive HIV-1 infection [33]. In this study, with the use of neuronal cells, we have observed that the presence of nicotine have significantly increased HIV infection which are in agreement with the previous reports observed in other types of cells [18,19,34]. Rock et al., reported that TGF-β1 may be involved in the effects of nicotine on enhanced HIV-1 expression in microglial cells [19]. Out of 84 synaptic plasticity genes analyzed, in this study, we have observed down-regulation of 28 synaptic plasticity genes (3–38 folds) and up-regulation (3–56 folds) of 8 genes in HIV infected cells. Our results indicate that HIV infection alters the expression of synaptic plasticity genes in neuronal cells which may contribute to the disrupted synaptic plasticity.

Nicotine treated SK-N-MC cells exhibit down-regulation in 23 synaptic plasticity genes (3–35 fold) and up-regulation in 16 genes (3–75 folds). Among these genes, 20 were commonly down-regulated in both nicotine-treated (alone) and HIV infected cells (alone). Recent studies have shown that nicotinic mechanisms influence forms of synaptic plasticity that are thought to underlie learning and memory [35,36]. Although nicotine is known to have neuroprotective effects, reports indicate that nicotine do not provide any evidence of the amelioration of the learning deficit (and slightly worsened performance during acquisition) observed in the behavioral HIV-1 transgenic (HIV-1Tg) rat model [37]. In few studies with nicotine exposure, improved attention, learning and memory processes were reported in both experimental animals and humans [16,17,38]. But the positive effect of nicotine on memory and attention is still a matter of debate in case of chronic nicotine exposure. In this study, we observed both positive and negative regulatory effects of nicotine on synaptic plasticity genes expression in neuronal cells after 7 days of chronic nicotine exposure.

**Figure 10:**
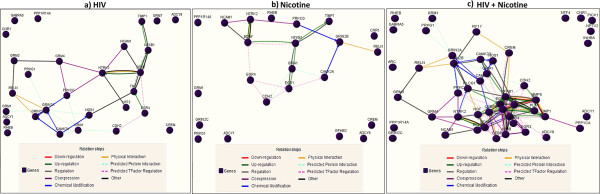
**Gene-Gene interaction network for human synaptic plasticity genes.** Human synaptic plasticity gene-gene interactions/relationships network was built using the GNCPro Gene Network Central for genes significantly down-regulated in SK-N-MC neuronal cells **a)** Infected with HIV; **b)** Nicotine treated; **c)** HIV infected cells in the presence of nicotine. The interactions are determined and color-coded by down-regulation, up-regulation, regulation, co-expression, chemical modification, physical interaction, predicted protein interaction, predicted TFactor regulation and other.

This is the first report to study the impact of nicotine on synaptic plasticity genes expression in HIV infected neuronal cells. HIV infected SK-N-MC cells in the presence of nicotine display a significant reduction in total 47 synaptic plasticity genes expression (3–84 folds) and significant up-regulation in only 4 genes expression (IGF1, NFKBIB, PPP2CA, RAB3A) (3–15 folds). All the genes down-regulated in nicotine/HIV infection alone are also down-regulated (few genes synergistically) in HIV infected cells co-treated with nicotine. The fold down-regulation ranges from 3–84 folds indicating increased neuropathogenicity in HIV infected cells treated with nicotine. Activation of immediate early transcription factors [immediate early genes (IEGs)] leads to the transcriptional activation of synaptic plasticity genes that presumably play an important role in the structural and functional neuronal changes involved in mammalian LTM [39,40]. Out of 30 immediate early response genes we have analyzed, 18 genes were significantly down-regulated in HIV infected SK-N-MC neuronal cells in the presence of nicotine, indicating transcriptional repression of other synaptic plasticity genes, therefore decreased LTM. Synaptic plasticity gene-gene interactions/relationships were built using the GNCPro Gene Network Central for genes significantly down-regulated in SK-N-MC neuronal cells Infected with HIV (Figure 10a); Nicotine treated (Figure 10b) and HIV infected cells in the presence of nicotine (Figure 10c).

Transcription factor NF- kappa-B (NF-kB) regulates spatial memory formation, synaptic transmission, and plasticity. Inhibition of NF-κB leads to decrease in forskolin-induced CREB phosphorylation [41]. In this study, along with down-regulation of NF-κB1, we have also observed up-regulation of NFKBIB that belongs to the NF-kappa-B inhibitor family, which inhibit NF-κB1 by complexing with, and trapping it in the cytoplasm that may further disrupt the synaptic plasticity in HIV infected neurons in the presence of nicotine.

NCAM1 is another highly down-regulated synaptic plasticity gene in the HIV infected neuronal cells in the presence of nicotine. NCAM1 is a multifunction transmembrane protein expressed in both neurons and glial cells with cell recognition properties involved in cellular migration, synaptic plasticity [42,43] and central nervous system development [42]. In this study, expression of NACAM1 was recovered with the use of vorinostat treatment.

Two other highly down-regulated synaptic plasticity genes in HIV infected neuronal cells and in combination with nicotine are the RHEB and TIMP1. While RHEB plays an important role in long term activity-dependent neuronal responses [44], TIMP1 is a candidate plasticity gene induced by seizures and by stimuli leading to long term potentiation. Down-regulation of these two genes may lead to the altered learning and memory.

We have also analyzed the dendritic spine density changes in HIV infected SK-N-MC cells co-treated with nicotine. We have got supporting results for the gene expression studies in SK-N-MC showing the significantly reduced spine density in the HIV infected cells co-treated with nicotine than the control, nicotine/HIV infected cells. We have also observed significantly decreased spine density in nicotine/HIV infected neuronal cells than the control cells indicating neurotoxicity in nicotine/HIV alone infected cells as well. Reduced cortical synaptic density has been reported in mild to moderate HAND patient’s autopsy samples [45]. Our study shows that nicotine severely affects spine density during HIV infection. This report provides evidence of increased neuropathogencity in HIV-infected neuronal cells exposed to nicotine and shows the induced synergistic decrease in synaptic plasticity genes expression and spine density than HIV/Nicotine alone infected cells. Among different nicotinic mechanisms influencing synaptic plasticity, role of overexpressed α7-nAChRs, which play a role in neurological disorders [46] was studied with nicotine/HIV. Chronic exposure to nicotine was reported to cause up-regulation of α7-nAChRs [47] and up-regulation of α7-nAChR has been reported as a contributor to the neurotoxicity associated with HIV infection as well [48]. Another important mechanism is the accumulation of excess glutamate in the extracellular space as a consequence of CNS trauma, neurodegenerative diseases, infection, or deregulation of glutamate clearance results in excitotoxicity. Most acute and chronic neuronal diseases, including HAND, have implicated this type of bystander pathology of excitotoxicity [49]. The presence of excess glutamate in the synaptic clefts activates glutamate gated ion channels and results in high levels of ion influx into neuronal cells allowing the over activation of downstream calcium ion-dependent effectors and signaling pathways, culminating in neuronal damage. Nicotine also reported to enhance the glutamate release [50,51]. Therefore, it may be possible that HIV in combination of nicotine may increase the excitotoxicity mediated by glutamate release.

Further, we have investigated the mechanisms attributed for the transcriptional and translational repression of synaptic plasticity gens in HIV infected cells and in combination with nicotine. We analyzed one of the important mechanisms, the role of HDAC2 in the transcriptional repression of synaptic plasticity genes. Histone acetylation is an epigenetic mark in the chromatin that favors gene expression and is modulated by the balance between histone acetyltransferase (HAT) and histone deacetylase (HDAC) enzyme activities, which add and remove, respectively, acetyl groups from histones. With the use of SKNMC, we showed significant up-regulation of HDAC2 in all the three study groups (1. HIV infected; 2. Nicotine treated; 3. HIV infected and co-treated with nicotine) indicating the possible role of HDAC2 in the transcriptional repression of synaptic plasticity genes in neurons. The evidence for HDAC2 in the negative regulation of memory and synaptic plasticity has been well documented [26,52] and the HDAC inhibitors especially class-I were reported as cognitive enhancers [53,54]. Loss of HDAC2 has been reported to improve the associative working memory and accelerated extinction learning [52]. Recently, Hanson et al., reported enhanced excitatory and reduced inhibitory synaptic transmission in HDAC2 knockdown single pyramidal neurons, whereas reduced excitatory and enhanced inhibitory synaptic transmission in HDAC2 overexpressed neurons [55]. Earlier, from our lab, we have reported the up-regulation of HDAC2 in human neurons in response to the HIV-1 Tat protein induced neurotoxicity [56]. Janus kinase (JAK)/signal transducer and activator of transcription (STAT) pathway is known to regulate the transcription of many genes [57-59]. Recently, the role of JAK/STAT pathway in synaptic plasticity has been reported [60].

Further we have used vorinostat, a potent, reversible HDAC inhibitor (both Class I and Class II) to inhibit HDAC2 expression in SK-N-MC cells infected with HIV and/or treated with nicotine. Interestingly, we observed increased HIV infection (latency disruption) in neuronal cells when treated with vorinostat at 1 μM concentration (no significant change in HIV infection in combined treatment with nicotine and vorinostat). Vorinostat reported to disrupt the HIV latency in resting CD4^+^ cells in vitro [61,62] and in vivo as well [63]. Our results suggest that vorinostat can disrupt the HIV latency in HIV infected CNS cells as well. Therefore, reactivation of latent HIV in the neuronal cells (by using vorinostat) along with antiretrovirals may provide a precise approach to treat and eradicate latent HIV infection in the brain. Vorinostat could also able to inhibit the HDAC2 up-regulation in neuronal cells infected with HIV and/or treated with nicotine. This results in a more relaxed chromatin configuration that allows the transcription of synaptic plasticity genes. Figure 1 shows the schematic representation of role of vorinostat in recovery of synaptic plasticity genes expression in neuronal cells infected with HIV and/or treated with nicotine. We have found that in all the groups (Nicotine, HIV infected, HIV + Nicotine), synaptic plasticity genes expression and spine density were recovered and many synaptic plasticity genes were further up-regulated when treated with vorinostat compared to the control cells. In vorinostat alone treated cells, 19 synaptic plasticity genes were significantly up-regulated and 4 genes (CREM, NFKB1, RHEB, TIMP1) were significantly down-regulated than control cells. Effect of down-regulation of these 4 genes in synaptic plasticity needs to be further evaluated. These results indicate that vorinostat positively regulate synaptic plasticity genes expression and spine density in HIV infected neurons and may be useful as a therapeutic agent in the treatment of HAND. Zolinza (Merck, Whitehouse Station, NJ), a prescription drug contains vorinostat is currently being used for the treatment of cutaneous T-cell lymphoma [64], multiple myeloma [65], mesothelioma [66]. Therefore, further studies to use vorinostat as a therapeutic agent against HAND and in nicotine users need to be studied.

In the down-stream, we have also analyzed one of the important mechanisms i.e. eukaryotic initiation factor 2α phosphorylation (at Ser51), which is responsible for the translational repression of synaptic plasticity genes. Due to partial phosphoryation of eIF2α, general translation is reduced, and ATF4 mRNA translation is augmented. As a consequence, expression of synaptic plasticity and memory-related genes is depressed (Figure 2). Decreased eIF2α phosphorylation reduces ATF4 mRNA translation and enhances general mRNA translation, thus facilitating the induction of synaptic plasticity genes expression, which leads to L-LTP and long-term memory (LTM) consolidation [67]. In our study, we have found increased eIF2α phosphorylation in two-study group of neurons (1. HIV infected; and 2. HIV infected and co-treated with nicotine). It is indicating that translation of synaptic plasticity genes will also be effected in the neuronal cells infected with HIV and in combined treatment with nicotine. To the best of our knowledge, till now, there are no reports of translational repression in HIV infected cells mediated by eIF2α phosphorylation. Therefore, further regulation of eIF2α phosphorylation in HIV infected neurons should be studied to control the translation of synaptic plasticity genes.

## Conclusions

There are few reports showing the cognitive impairment in heavy smokers [68-71]. Effects of cigarette smoking on cognitive disorders in HIV infected patients are scanty [23,72]. This is the first study in analyzing the molecular mechanisms behind the increased risk of HAND in HIV infected nicotine users. This study concludes that HIV infected nicotine users are more prone to develop HAND symptoms compared to HIV infected patients who are not nicotine addicts, due to the HDAC2 induced transcriptional repression. These negative regulatory effects on synaptic plasticity genes expression and spine density may be reversed by using the vorinostat (class I and II HDACs inhibitor). Vorinostat may be a useful therapeutic to inhibit the neurotoxic effects on synaptic plasticity in HIV infected nicotine abusers.

## Materials and methods

### Cell culture and virus

SK-N-MC neuronal cells were obtained from ATCC (Cat # HTB-10). HIV-1Ba-L (clade B, macrophage-tropic [R5] virus) was obtained through AIDS Research and Reference Reagent Program, Division of AIDS, NIAID, NIH (NIH AIDS Reagent Program Cat. # 510).

### HIV-1 infection and/or nicotine treatment of SK-N-MC cells

SK-N-MC neuronal cells were infected with HIV-1 using the previously described protocol [13,73-75] with slight modifications. Briefly, SK-N-MC cells (1×10^6^ cells) were cultured overnight in T-75 flasks using complete minimum essential medium. The cells were activated by treating with polybrene (10 μg/ml) for 8 hrs before the infection. The cells were infected with TCID50 of HIV-1 and/or treated with 1 μM-1 mM nicotine (Sigma, Cat # N3876) for 7–10 days under same experimental conditions. In every second day, 2 ml of fresh medium (with/without nicotine) was added to the culture. Controls cells (without HIV-1/Nicotine) were included in the set-up of all experiments.

### Inhibition of HDAC2 up-regulation

SK-N-MC cells were infected with HIV and/or treated with nicotine as explained above. On the 5th day of HIV infection and/or nicotine treatment, SK-N-MC cells were treated with 0.1-10 μM vorinostat. After total 7 days of HIV infection and/or nicotine treatment, expression of HDAC2 was analyzed by qPCR and western blot.

### mRNA and protein extraction; and first strand cDNA synthesis

After 7 days of HIV infection and/or co-treatment with optimum concentration of nicotine (100 μM)/Vorinostat (1 μM), SK-N-MC cells were harvested and the pellet was used for the mRNA isolation using illustra triplePrep Kit (GE Healthcare Life Sciences, UK; Cat # 28-9425-44) and on-column DNase treatment step was also performed in the procedure. Purity and concentration of the RNA was measured by microspot RNA reader (Synergy HT Multi-Mode Microplate Reader from BioTek, US) and RNAs with an OD260 nm/OD280 nm absorbance ratio of at least 2.0 were used for PCR array. This mRNA was also used for the long terminal repeat (LTR) and HDAC2 gene expression using RT-qPCR. One microgram of RNA from all the control and test groups (1. Uninfected/untreated; 2. Nicotine; 3. HIV; 4. HIV + Nocotine; 5. Vorinostat; 6. Nicotine + Vorinostat; 7. HIV + Vorinostat and 8. HIV + Nicotine + Vorinostat) was used for the first strand cDNA synthesis using SABiosciences’s RT2 First Strand Kit (Cat. # 330401) as per supplier’s protocol. Genomic DNA elimination step was performed prior to reverse transcription. With the use of illustra triplePrep Kit, from the same set of samples, protein was precipitated and preceded for SDS-PAGE after the estimation of protein concentration.

### QPCR of viral transcripts in the presence of nicotine

The cDNA for m-RNA isolated from all control and test groups (as mentioned above) was synthesized using the high-capacity reverse transcriptase cDNA kit (Applied Biosystems, Cat # 4368814) to perform qRT-PCR by Taqman gene expression assay as described by manufacturer’s protocols. A quantitative HIV-1 DNA protocol (LTR real-time PCR) was used to analyze the viral transcripts in HIV infected SK-N-MC cells and in combination with nicotine and vorinostat. The following previously published [76] primers and probes were used: LTR U5/R-sense-5′-GGCTAACTAGGGAACCCACTG-3′ and antisense-5′-CTGCTAGAGATTTTCCACACTGAC-3′, probe 5′-FAM-TGTGTGCCCGTCTGTTGTGTG-TAMRA-3′. Values were normalized against GAPDH.

### Expression of HDAC2 in SK-N-MC neuronal cells infected with HIV and/or treated with nicotine/vorinostat combination

In the SK-N-MC cells infected with HIV and/or treated with nicotine/vorinostat combination (as explained above), expression levels of HDAC2 was measured by qPCR with the use of Taqman assay (Assay ID # Hs00231032_m1) and by western blot using anti-HDAC2 antibody (Millipore cat # 07–222).

### Human synaptic plasticity RT^2^ profile PCR array

Synaptic plasticity gene expression profiling was done in all the neuronal cell control and test groups (all the groups as mentioned above) using 96 well format RT^2^ Profile PCR Array human Synaptic Plasticity kit (SABiosciences, Cat. # PAHS-126A-2) using Stratagene Mx3000p qRT-PCR instrument. The human Synaptic Plasticity RT^2^ Profiler PCR Array interrogates 84 genes related to the human synaptic plasticity. This kit was chosen because it includes diverse genes important in the human synaptic plasticity, including Immediate-Early Response (n = 30), Late Response (n = 2), Long Term Potentiation (n = 28), Long Term Depression (n = 21), Cell Adhesion (n = 9), Extracellular Matrix & Proteolytic Processing (n = 5), CREB Cofactors (n = 10), Neuronal Receptors (n = 19), Postsynaptic Density (n = 15), as well as other genes involved in the synaptic plasticity (n = 2). Few genes have role in multiple functions listed above. Relative abundance of each mRNA species was assessed using RT^2^ SYBR Green/ROX PCR Master mix (SABiosciences, Cat # 330520) and aliquoted in equal volumes (25 μl) to each well of the real-time PCR arrays. The real-time PCR cycling program (as indicated by the manufacturer) was run on a Stratagene Mx3000p qRT-PCR thermal cycler. The threshold cycle (Ct) of each gene was determined by using the Stratagene MaxPro software. CT data were uploaded into the data analysis template on the manufacturer's website (http://pcrdataanalysis.sabiosciences.com/pcr/arrayanalysis.php). The relative expression of each gene in each test group was compared with the expression in control cells and it was calculated on the website using the ΔΔCT method with five housekeeping genes as controls. Controls are also included on each array for genomic DNA contamination, RNA quality, and general PCR performance.

### Measurement of spine density

#### DiI staining

Established protocol to stain the neuronal cells and measurement of the spine density (in all the control and test group neuronal cells) was used with few modifications [13,74,77-81]. In brief, SK-N-MC cells were grown in Eagle’s minimal essential medium (MEM) containing 10% fetal bovine serum (FBS), 5 mM sodium pyruvate, 100 units/ml penicillin, 100 mg/ml streptomycin and retinoic acid at 37°C with 5% CO2. SK-N-MC cells were grown onto 22 mm × 50 mm glass coverslips placed in a petri-dish. Cells were treated with polybrene for 8 hrs followed by addition of HIV and/or nicotine. On the 5th day of HIV infection and/or nicotine treatment, vorinostat (1 μM) was added to the respective test groups. After 7 days of infection and/or treatment with nicotine, cells were fixed in 4% Formaldehyde in PBS for 30 min at RT. The fluorescent membrane tracer 1, 1'-Dioctadecyl-3, 3,3’,3’-tetramethylindocarbocyanine perchlorate (DiI) at 5 μg/ml in PBS concentration was directly added onto the fixed cultures and allowed to incubate for 90 min at RT. The stained coverslips were placed at 4˚C in small petri dishes containing PBS and allowed for overnight for the transport of dye. Cover-slips were mounted on the slides with Prolong®Gold antifade reagent (Invitrogen) before proceeding for confocal microscopy.

#### Confocal microscopy

Confocal images were obtained using TCS SP2 Confocal Laser Scanning Microscope (Leica Microsystems, Germany) at 488 nm (100%) illusion of an argon-ion laser using 60X oil immersion objectives with high numeric aperture and 2.5X confocal electronic zoom settings to visualize individual cells and dendrites. Twenty Optical serial sections of 0.14 μm/section (~2.8 μm total) through the cells were reconstructed to yield complete “two dimensional” images of individual cells in focus.

### Western blot assay

For SDS-PAGE, similar amounts of control and test group SK-N-MC cellular protein, typically 40 μg per lane were used. All the protein samples were separated by using Any KD Mini-Protean TGX precast Gels (Bio-Rad, Cat # 456–9034). Proteins were transferred to nitrocellulose membranes, and the quality of protein measurement, electrophoresis, and transfer was checked by staining with Ponceau S. Membranes were blocked with 5% skimmed milk in TBS-T (20 mM Tris buffer, pH 7.5, 0.5 M NaCl, 0.1% Tween 20) for 1 hr at room temperature and incubated at 4°C overnight in the primary antibody diluted in 2% skimmed milk in TBS-T. The primary antibodies used were as follows: anti-HDAC2 antibody (Millipore cat # 07–222, 1/5000); rabbit anti-phospho-eIF2α (Ser51) antibody (Cat # 9721, 1/1000), rabbit anti-eIF2α antibody (Cat # 9722, 1/1000) from Cell Signaling (Beverly, MA). Subsequently, blots were washed in TBS-T (4 times, 10 min each) and incubated for 1 hr at room temperature in horse radish peroxidase-goat anti-rabbit antibody (Promega, Cat # W401B) diluted 1/2500 in 2% skim milk in TBS-T. After additional washings, protein bands were detected by chemiluminiscence using SuperSignal West Pico Luminol/Enhancer (Thermo Scientific, Cat # 1856136) and SuperSignal West Pico substrate (Thermo Scientific, Cat # 1856135). Band density was measured by using ImageJ software.

### Data analysis

In the expression studies, a gene was considered differentially regulated if the difference was ≥3 fold in comparison with the control. Experiments were performed at least three times and the values obtained were averaged. All the results are expressed as mean ± standard error of the mean. Statistical analysis of two groups was performed by Student’s t test, while more than two groups were analyzed using one way ANOVA followed by Bonferroni’s multiple comparison test. Differences were considered significant at p ≤ 0.05. If the combined effect observed is significantly greater than the expected (additive) effect, it was considered synergism [82]. Data analysis was performed with the Statistical Program, GraphPad Prism software (La Jolla, CA).

ImageJ software program was used to quantify DiI-labeled cells. Dendritic segments were chosen randomly from the apical and basal regions and at least one soma’s length away from the cell soma.

## Competing interests

The authors declare that they have no competing interests.

## Authors’ contributions

VSRA, MN, and SKS conceived and designed the experiments. VSRA performed the experiments. VSRA, SPK, VS, KRVK, ST, HD, AR and OH analyzed the data. MN contributed reagents/materials/analysis tools. VSRA, AR, SPK and KRVK prepared the manuscript. All authors read and approved the final manuscript.
